# Can international health programmes be sustained after the end of international funding: the case of eye care interventions in Ghana

**DOI:** 10.1186/1472-6963-14-77

**Published:** 2014-02-19

**Authors:** Karl Blanchet, Philip James

**Affiliations:** 1 International Centre for Eye Health, Clinical Research Department, London School of Hygiene and Tropical Medicine, London, UK; 2 School of Environment and Life Sciences, University of Salford, Salford, UK; 3 London School of Hygiene and Tropical Medicine, Keppel Street, London WC1E 7HT, UK

**Keywords:** Sustainability, Program, Diffusion of innovation, Administrator, Hospital, Analysis, Decision, Eye care

## Abstract

**Background:**

There is general agreement amongst major international policy makers that sustainability is a key component of health interventions in developing countries. However, there is little evidence on the factors enabling or constraining sustainability. Diffusion of innovation theory can help explain how the continuation of activities is related to the attributes of innovations. Innovations are characterised by five attributes: (i) relative advantage; (ii) compatibility; (iii) complexity; (iv) triability; and (v) observability. An eye care programme was selected as a case study. The programme was implemented in the Brong Ahafo region of Ghana and had been funded over a ten-year period by an international organisation.

**Methods:**

Sustainability in the study was defined as the level of continuation of activities after the end of international funding. Measuring the continuation of activities involved checking whether each eye care activity continued (i.e. out-patient consultation, cataract surgery, outreach, school health, and statistics) or was interrupted after the end of Swiss Red Cross funding the 11 district hospitals where the programme was implemented.

**Results:**

The results showed a relationship between the level of sustainability and the attributes of every activity. The activities with the lowest score for the attributes were less sustained. School health screening was the least sustained activity after the end of international funding. This activity also held the smallest score in terms of attributes: they were the most incompatible and most complex activities, as well as the least triable and observable activities, amongst the four district activities. In contrast, compared to the three other district activities, facility-based consultations were more likely to be routinised because they were perceived by the hospital managers as very compatible, and not complex.

**Conclusions:**

Using diffusion of innovations theories can help predict the sustainability of specific activities within a health programme. The study also highlighted the need for disentangling the various components of a health programme in order to identify which activities are more likely to be continued within a health system. The same methodology could be used in a different setting and could help predict which innovations are more likely to be adopted and maintained over time.

## Introduction

There is general agreement amongst major international policy makers that sustainability is a key component of health interventions in developing countries [1-6]. The term "sustainability", defined in broad terms as the continuation of benefits [7], has become a buzzword in international development over the past two decades [8-10]. It has generated rich, enthusiastic debates, and innovative theories beyond the sphere of international health, and has even led to the creation of "sustainability science" [11]. Everything must now be sustainable, not only the environment, but also cities, agriculture, livelihoods, and health. However, the concept of sustainability has been overused [12] and, as a result, means different things to different people [13-15]. The absence of a clear and operational definition of sustainability has also generated ambiguous and vague methods for measuring sustainability in public health [15,16]. A challenge for policy-makers and researchers is to translate the concept of sustainability into concrete indicators, and generate evidence on the factors constraining and enabling sustainability [13,17].

The objective of the study carried out in Ghana, commencing eighteen months after the end of international funding provided by the Swiss Red Cross, was to identify sustainability factors, focusing on the characteristics of health activities implemented in district hospitals. For the first time in health systems research, the attributes of activities in a health programme implemented in a low or middle income country were described using diffusion of innovations theories; and a relationship was established between the level of continuation of activities (sustainability) and these attributes.

### Diffusion of innovations theories

In the late 1990s, a few scholars proposed introducing systems thinking in international health in order to take local perspectives and contexts into account [18,19]. The introduction of systems thinking and complexity science in international development is reflective both of the failure of international donors and NGOs to deliver long term benefits to the population. Health systems are viewed as complex systems [20-23]. Complex systems are systems with a high number of elements or actors that interact with each other in ways that are not always predictable following the introduction of an innovation (e.g. a new health intervention) [21,24,25]. Introducing an innovation into a complex adaptive system (e.g. a district hospital) can produce extensive changes in various socio-technical aspects of the system, including tasks of individuals, relationships between actors or management mechanisms [26,27]. An innovation is defined in this paper as "an idea, practice or object that is perceived as new by an individual or other unit of adoption" [28 Page 12]. The process generated by the introduction of an innovation is described by Rogers [28] as an "innovation-decision process".

Diffusion of innovation theory can help explain how the continuation of activities is related to the attributes of activities as innovations. Beyond the description of an innovation as a newness, Rogers [28] showed that innovations are characterised by five attributes:

(i) relative advantage-individuals assess innovations by comparing the expected advantage of the new initiative with the benefits provided by the previous one that it replaced;

(ii) compatibility-an innovation is perceived as compatible when the new idea or technology introduced by the innovation is consistent with the mandate of the adopters or the adopting system and does not require significant modifications from the adopters [29,30];

(iii) complexity-the perceived difficulty in understanding a new idea or using a new technology. A complex innovation can also be an intervention which involves a high number of actors [30-32];

(iv) triability-the notion that an innovation can be tested on a small scale [33].; and

(v) observability-the degree to which the results of the innovation are visible [28,31,33].

An eye care programme was selected as a case study. The programme was implemented in the Brong Ahafo region of Ghana and had been funded and supported over a ten-year period by an international organisation. International support terminated in December 2006, 18 months before the start of the study.

## Background

### Study site: Ghana and the brong ahafo region

Ghana has a population of approximately 20 million, distributed across 10 administrative regions and 138 districts. Ghana enjoys a measure of relative economic stability, with an average annual gross domestic product growth rate of 5.6% between 1999 and 2009 [34]. The level of poverty decreased by almost 50% between 1992 and 2006 but remains high with 28% of the Ghanaian population living in 2006 with less than $1 per day [35].

In terms of eye diseases, Ghana has the second highest prevalence of glaucoma in the world: 8.5% of people over 40 years old. Cataract is the highest cause of blindness in Ghana [36]. Eye care access is unequally distributed within Ghana and there are inequities between populations living in rural areas and in urban areas, as well as between the north and south of the country [37]. For example, 39 the ophthalmologists (i.e. 75% of the total number of ophthalmologists working in Ghana) live in the south of the country, on the coast, and only 5 ophthalmologists work in the four regions of the north (i.e. Brong Ahafo, Northern Region, Upper West and Upper East)–these four regions also have the poorest health indicators of the country [38]. Since the 1990s, there has been considerable emphasis in Ghana on improving equity of access to health care. A national health insurance scheme has been introduced to improve access for the poor by reducing the financial barriers to accessing services [37].

The Ministry of Health regulates the whole health sector, and health services are delivered by two agencies: Ghana Health Service in charge of public health facilities and the Christian Health Association of Ghana (CHAG) in charge of faith-based health facilities. In Ghana, 42% of health facilities are managed by CHAG [39].

The Brong Ahafo region, with a population in 2009 of 2 million [40], is in the centre of the country. Brong Ahafo, with an urbanisation rate of 37.4%, is the fourth most urbanized region of Ghana (amongst the ten regions of the country). With the exception of the Sunyani District, the major urban centre of the region, agriculture is the major source of income for households in all districts. The Brong Ahafo region is divided in 18 health districts. In 2009, Brong Ahafo had one doctor for every 17,000 people: the fourth best ratio in the country [38]. Brong Ahafo is also the region with the best outpatient attendance per capita [38]. In Ghana, eye care services are provided in the 19 district hospitals and the regional hospitals. Both ophthalmologist and ophthalmic nurses are involved in the delivery of curative eye care services.

### The eye care programme

Prior to 1996, no eye care service existed in the Brong Ahafo. There was no ophthalmologist or ophthalmic nurse in the whole region [41]. Patients had to travel between two and five hours (one way) to Kumasi, the regional capital of the neighbouring Ashanti region to access an ophthalmologist. The eye care programme implemented by the Swiss Red Cross, the Ghana Red Cross, and the Ghana Health Service was the first initiative in eye care in the Brong Ahafo region. The eye care programme selected 12 out of the 19 districts of the Brong Ahafo region in order to target their resources. One ophthalmologist and 12 ophthalmic nurses were funded as part of the programme. The ophthalmologist operated in the regional hospital, in Sunyani, and conducted outreach surgeries in district hospitals. The majority of ophthalmic nurses were posted in district hospitals (one ophthalmic nurse per district hospital) and conducted consultations in district hospitals. They contacted the ophthalmologist when 20-30 patients in need of routine cataract surgery had been identified and referred complicated cases to the regional hospital. Ophthalmic nurses also conducted outreach activities (consultations in villages) and school health screening (assessment of the visual acuity of pupils in schools). In terms of financing, all the direct costs of the activities were funded by the Swiss Red Cross (i.e. medical consumables for surgeries, fuel and per diem for the outreach activities including school health screening). Eye care staff were all employed and paid by their hospital. From a patient perspective, user fees were only paid for facility-based activities (consultations and cataract surgeries). All outreach activities (outreach consultations and school screening) were delivered free of charge for the patient.

The objective of the programme was to increase uptake of eye care services in the Brong Ahafo region. The objective of the programme remained stable over the course of the programme (i.e. between 1996 and 2006) [41,42].

## Methods

The definition of sustainability adopted in the study was based on how the eye care programme was designed and how the information system of the programme was structured [43]. Sustainability in the research was defined "as the level of continuation of activities after the end of international funding" (after Honadle and Sant [7]). Sustainability was measured by comparing the number of outputs per activity before and after the end of international funding (18 months after the international funding ceased) (e.g. ‘surgery activities’ were measured by the number of cataract surgeries performed).

### The continuation of activities

The units of analysis were the district hospitals of the Brong Ahafo region where the eye care programme took place. The eye care programme was implemented in 11 of the 19 district hospitals in the Brong Ahafo region. The level of sustainability was measured in all of these 11 district hospitals. All reports (statistics, activities, evaluations, and budgets) produced during the ten years of the eye-care programme were collected. During the course of the study, additional documents (e.g. health facility statistics, reports from regional authorities, minutes of management committee meetings, mission reports from the Swiss Red Cross, national policies) were collected and analysed. Documentary research was used for data triangulation and theory triangulation [44], and also helped raise new research questions and identify new informants.

Measuring the continuation of activities involved checking whether each eye care activity continued (i.e. out-patient consultation, cataract surgery, outreach, school health, and statistics) or was interrupted after the end of Swiss Red Cross funding [45]. In other words, activities that were functioning in December 2006 were compared with the activities that were still in place in July 2008 (the date of the assessment). An activity was defined as "functioning" when the number of outputs from that activity was greater than zero (an output is defined as the number of health care services produced by a health facility: e.g. number of consultations, number of cataract surgeries) (see Table 1). In addition, an activity was considered "sustainable" if the activity was functioning at both points in time (i.e. the number of outputs was greater than zero). Similarly, the programme was defined as "sustainable" if every activity in every district hospital was functioning at both points in time. In other words, if one activity was interrupted in one hospital during the second quarter of 2008, the programme was considered "not sustainable".

**Table 1:** List of indicators used to measure the level of sustainability of the programme at district level

**Activity**	**Indicator**	**Source of information**
Outpatient consultations	Number of patients consulted at the eye clinic of the district hospital	Medical records and statistics reports in hospitals
Cataract surgery	Number of cataract surgeries performed at the district hospital	Medical records and statistics reports in hospitals
Outreach consultations	Number of people consulted in communities (villages) during outreach consultations	Medical records and statistic reports in hospitals
Screening of pupils in schools	Number of pupils screened by the nurse at school level	Medical records and statistics reports in hospitals
Quarterly statistics reports	Availability of quarterly statistics reports	Statistics reports in hospitals

### Attributes of innovations

A long list of sustainability indicators was developed in 2009 based on a review of the literature on innovations. Ten key informants involved in the eye care programme in Ghana at senior level in the management of the eye care services and policies reviewed and edited this long list to ensure that each indicator was relevant to the local context. A consensus meeting was organised during two days with all the key informants to agree on a final list of common indicators. Data relevant to each indicator was then collected and checked through consultation with key informants.

(i) Relative advantage

 The first characteristic described was the relative advantage new eye care activities could provide compared to previous eye care activities. The activity of the eye programme was compared to an eye care-related activity that was present at the hospital before the implementation of the eye care programme.

(ii) Compatibility

 Criteria were defined in order to measure levels of compatibility (Table 2).

(iii) Complexity

 Complexity indicators (Table 3) were developed.

(iv) Triability

 The indicators for triability are presented in Table 4:

(v) Observability

 The indicators for observability are shown in Table 5.

**Table 2:** Criteria for measuring the compatibility of an activity

**Compatibility**				
**Very high**	**High**	**Medium**	**Low**	**Very low**
Fully coherent with mandate & no modification required	Almost coherent with mandate & no modification required Or Fully coherent with mandate & insignificant modifications required	Almost coherent with mandate & insignificant modifications required	1. Not coherent with the mandate & insignificant modifications required Or 2. Almost coherent with the mandate & significant modifications required	Not coherent with the mandate &significant modifications required

**Table 3:** Criteria for measuring the complexity of an activity

**Complexity**				
**Very high**	**High**	**Medium**	**Low**	**Very low**
Engagement of actors outside the hospital working at two different levels & utilisation of technology not usually available at the service level	Engagement of actors outside the hospital all working at the same level & utilisation of technology not usually available at the service level	Engagement of actors outside the hospital all working at the same level & no utilisation of technology not usually available at the service level	Engagement of only one actor outside the hospital & no utilisation of technology not usually available at the service level	No engagement of actors outside the hospital & no utilisation of technology not usually available at the service level

**Table 4:** Criteria for measuring the triability of an activity

**Triability**				
**Very high**	**High**	**Medium**	**Low**	**Very low**
Every sub-activity of the activity can be independently tested on a small scale	Most sub-activities (70%) of the activity can be independently tested on a small scale	Half of sub-activities of the activity can be independently tested on a small scale	Most sub-activities (70%) of the activity cannot be independently tested on a small scale	The sub-activities of the activity cannot be independently tested on a small scale

**Table 5:** Criteria for measuring the observability of an activity

**Observability**				
**Very high**	**High**	**Medium**	**Low**	**Very low**
The activity consists of treatment with immediate effect (within one hour) on the patient	The activity consists of treatment with rapid effect (within one day) on the patient	The activity consists of treatment with slow effect (within days) on the patient	The activity consists of treatment with very slow effects (within weeks) on the patient	The activity does not provide any treatment

In brief, the level of continuation of each programme activity (i.e. out-patient consultation, cataract surgery, outreach, school health, and statistics) was measured. Then, for each programme activity, the indicators of the five attributes were measured and are presented in the following section. In-depth interviews were conducted by the first author to understand the reasons for continuation or interruption of activities. 51 interviews were carried out in 2009 with Officers at the Ministry of Health, regional and district health authorities, district hospital managers and health staff, Swiss Red Cross Officers and community members. All the ophthalmic nurses in health facilities, the regional ophthalmologist and the regional Health Officers in charge in 2006 of eye care service delivery were still in place at the time of the study in 2008 and 2009.

### Limitations

One issue concerned the level of accuracy of medical records. As observed by the first author during data collection, medical records collected in district hospitals were not always coherent or complete. This was obvious when figures were missing or when the writing style in the books changed. However, the level of detail required by the investigator only concerned the volume of activities. No data was collected about type of diseases, limiting errors of misclassification.

The other challenge met by the investigator was that medical records were not always conserved or maintained in good condition: they were recorded in books and kept on shelves, and a few old books were missing and could not be found at the hospital. In addition, some activities were not correctly reported; for example, at the start of the programme in 1996, ophthalmic nurses did not differentiate between facility-based consultations and outreach consultations. Record keeping dramatically improved from the year 2000, when the Swiss Red Cross put a lot of effort into reporting and requested that nurses systematically record patients in their books categorised by activity.

The investigator was assisted by the ophthalmic nurse in charge of the eye clinic to fill in missing information when a figure was not clearly written or when records were absent. All ophthalmic nurses responded positively to the requests of the investigator and provided support by researching medical records in archives or asking the administrator of the hospital to provide the correct figures when available.

Data on hospital admissions was triangulated in order to verify the veracity of the following three sources: i) daily records completed by the ophthalmic nurse following each consultation); ii) quarterly records completed by the ophthalmic nurse but submitted to the Swiss Red Cross every three months; iii) annual records derived from annual hospital reports. The investigator compared the two sources of information. When there was a difference between the two sources of data, the figures were not considered valid and were not reported.

Ethical clearance received from the Ministry of Health of Ghana and the London School of Hygiene and Tropical Medicine.

## Results

### Level of continuation of health activities

A comparison between the different levels of continuation of eye health activities is described between the month when international funding ceased and the initiation of the study, i.e. between December 2006 and July 2008. The level of continuation of the five district activities (out-patient consultation, cataract surgery, outreach, school health, and statistics) in the Brong Ahafo region was measured in every district hospital containing an eye clinic with the permanent presence of an ophthalmic nurse to manage the clinic.

Tables 6 and 7 illustrate which activities still functioned in July 2008 for each hospital. All the district activities were functioning in December 2006.

**Table 6:** Programme activities that continued (✓) or stopped (×) in every district in July 2008

**District hospitals**	**Out-patient consultation**	**Cataract surgery**	**Outreach**	**School health**	**Statistics**
1. Atebubu	✓	**×**	**×**	**×**	✓
2. Bechem	✓	**×**	**✓**	**×**	✓
3. Dormaa	✓	✓	**×**	**×**	✓
4. Drobo	✓	✓	**×**	**×**	✓
5. Goasso	✓	✓	**×**	**×**	✓
6. Kintampo	✓	**×**	**×**	**×**	✓
7. Nkoranza	✓	✓	✓	**×**	✓
8. Sampa	✓	**×**	**×**	**×**	✓
9. Techiman	✓	✓	✓	**×**	✓
10. Wenchi	✓	✓	✓	✓	✓
11. Yeji	✓	**×**	**×**	**×**	✓

**Table 7:** Number and percentage of district hospitals (n = 11) where the district activities were maintained in July 2008

	**Out-patient consultation**	**Cataract surgery**	**Outreach**	**School health**	**Statistics**
Number of district hospitals where the activity continued	11	7	4	1	11

In order to identify whether there were differences in continuation between activities, a scoring system was applied where a score of 1 was allocated for each activity that was being conducted in July 2008 and a score of 0 was allocated for each activity that was not being conducted in July 2008. An equal weighting was given for a score of 1 or 0, as there is no evidence in literature that one attribute has more impact than another one. This enabled calculation of the percentage of hospitals conducting each activity in July 2008. It was also examined whether the activity output had progressively decreased.

The only two activities that were maintained in all eleven hospitals between December 2006 and July 2008 were outpatient consultations and compilation of quarterly statistics. Cataract surgeries were continued in 58% of the hospitals (i.e. 7 hospitals). However, outreach activities and school health activities continued in a limited number of hospitals. Outreach activities were maintained in 33% of hospitals (i.e. 4 hospitals) and only one hospital continued school health between December 2006 and July 2008. These findings indicate that the level of maintenance of activities varied between the different types of activities.

### Relative advantage

The relative advantage attribute is a comparison between the current innovation and past innovations. Were the current eye programme activities an added value compared to the precedent programme? The eye care programme was the first initiative of its sort in the Brong Ahafo region. No eye care services were available before the implementation of the eye care programme. As a result, the relative advantage of the eye care programme compared to another type of programme was perceived by hospital managers as very high for every district activity of the programme (Table 8).

**Table 8:** Relative advantage of every district activity

**Relative advantage**	**Very high**	**High**	**Medium**	**Low**	**Very low**
Facility-based consultations	✓				
Cataract surgery	✓				
Outreach activities	✓				
School health	✓				

As shown in previous studies [30,46], the perceived relative advantage of an activity does not guarantee its maintenance. This study confirmed this finding. The relative advantage attribute did not explain differences between activities in terms of continuation.

### Compatibility

An innovation was defined as compatible when a new idea or technology introduced by the innovation was coherent with the mandate of the adopters and did not require significant modifications from the adopters [29,30]. The eye care programme was developed in collaboration with the Ministry of Health of Ghana and its implementing agencies: the Ghana Health Service and the Christian Health Agency of Ghana. In terms of compatibility, the general eye care programme followed the policies and values of the Ministry of Health of Ghana, and international policies and guidelines developed by the World Health Organisation [47].

However, there were nuances in function of the activity concerned, which may be explained by considering the structure of the health system in Ghana. The mandate of district hospitals was to deliver facility-based services and serve the population living in their catchment area (i.e. the district). Every facility-based activity, such as consultations and cataract surgeries, was considered by hospital managers as fully compatible with the mandate of a Ghanaian district hospital. These two activities were fully in compliance with the mandate of district hospitals and no modification was required from the district hospitals to adjust their structure to deliver these activities. As such, facility-based consultations and cataract surgeries were perceived by hospital managers to be highly compatible innovations.

Hospital managers acknowledged that confusion existed over who should be responsible for the management of outreach and school health activities. One hospital manager explained: "outreach activities should have been carried out by the district authorities, not by the hospitals. They are the ones in charge of all the health interventions that take place outside the walls of this hospital. However, they never took the initiative and our staff had to conduct the outreach consultations". As another hospital manager described it, "outreach activities and school health activities had never been seen as a problem as long as the costs of these activities were fully covered by the international organisation. As soon as the international organisation ceased its financial support, it became unclear for the health actors who should continue these activities, the hospital or the district authorities".

The lack of clarity regarding the outreach activities and school health activities created conflicts between two distinct entities: the district health management teams and the district hospital. In the Ghanaian health system, district hospitals are not accountable to the district health management teams, even when district hospitals are managed by the same public agency, in this case, Ghana Health Service. The district health management teams are in charge of all the activities conducted at the primary health care level of the health system (i.e. any activity conducted outside the district hospital). Outreach consultations and school health activities were perceived by hospital managers as inconsistent with the mandate of the district hospitals because they took place outside the district hospital. They also required slight adaptations from district hospital teams as described by one ophthalmic nurse: "Outreach activities and school screening introduced new ways of working at the hospital. We had to contact headmasters or local authorities to offer our services. We then organised trips in collaboration with these people. This was very unusual for us. We were used to wait for the patient to come to the hospital. Now we had to do the inverse". Outreach and school activities were described as almost inconsistent with the mandate of hospitals and required adjustments from hospitals, which corresponded to a low level of compatibility (Table 9).

**Table 9:** Level of compatibility of every district activity

**Compatibility:**	**Very high**	**High**	**Medium**	**Low**	**Very low**
Facility-based consultations	✓				
Cataract surgery	✓				
Outreach activities				✓	
School health				✓	

Facility-based consultations and cataract surgeries were highly compatible with the mandate of district hospitals as the two other activities, outreach activities and school health, had a low level of compatibility with the mandate of hospitals.

### Complexity

Complexity was defined in the context of the study not in terms of technological or clinical complexity but rather in terms of number of players involved in the implementation of one activity: the higher the number of actors, the more complex the activity. The two benchmarks chosen for the study to define the different levels of complexity were, for the least complex level, the facility-based consultations that did not require the involvement of additional actors outside hospital staff and did not utilize additional technology outside the standard equipment already available in district hospitals. Outreach activities and school health, as the most complex activities amongst the four district activities, required the engagement of a high number of different actors from various spheres of society (e.g. community leaders, sub-districts clinics) and the utilisation of technology that is considered as expensive and rare in the Ghanaian health system: motorbikes. The engagement of various actors working at different levels of the health system, and utilisation of technology is a description of a highly complex technology.

At the district level, the level of complexity of each activity was determined by cross comparison between activities (Table 9, below). Cataract surgeries were defined as highly complex innovations, as they demanded the engagement of players working at two different levels of the health systems: the ophthalmologist at the regional level, the ophthalmic nurse and the hospital manager at the district level. The technology used for cataract surgery did not require additional investments from hospitals.

Outreach activities and school health screening were perceived as highly complex interventions, followed by cataract surgery, highly complex innovation (Table 10). The facility-based consultations were considered to be activities with a low level of complexity.

**Table 10:** Level of complexity of every district activity

**Complexity:**	**Very high**	**High**	**Medium**	**Low**	**Very low**
Facility-based consultations					✓
Cataract surgery		✓			
Outreach activities	✓				
School health	✓				

### Triability

Triability refers to the notion that an innovation can be tested on a small scale [33,48]. The four eye care activities required specific specialised eye care staff (ophthalmologist and ophthalmic nurse). None of the activities could be tried by the hospitals using their usual health staff (Table 11 below). This constraint made the triability of the eye care activities very limited. None of the four activities could be tried by the hospital managers without the recruitment of specialised eye care staff, which was an expensive investment and did not give the latitude to hospital managers to abandon the innovation if they did not deem it to be worthwhile continuation.

**Table 11:** Triability of district activities

**Triability:**	**Very high**	**High**	**Medium**	**Low**	**Very low**
Facility-based consultations					✓
Cataract surgery					✓
Outreach activities					✓
School health					✓

### Observability

Observability is the degree of visibility of the results of an innovation [28,31,33]. Ophthalmology and more specifically cataract surgery presents the great advantage of producing rapid and visible results. After cataract surgery, the effect on blindness is immediate and observable by the patient him/herself and his/her relatives. In other areas of medicine, treatment can take effect several days after treatment. Cataract surgery has highly observable effects as a patient can recover sight as soon as an hour after the operation. Facility-based and outreach consultations both have a medium level of observability as the effects on the patients could sometimes take weeks before experiencing any improvement after medication. The activity with the lowest level of observability was school health as no direct effect of the health of the patients’ pupils could be seen after school screening (Table 12).

**Table 12:** Observability of every district activity

**Observability:**	**Very high**	**High**	**Medium**	**Low**	**Very low**
Facility-based consultations			✓		
Cataract surgery	✓				
Outreach activities			✓		
School health					✓

Cataract surgery was the only activity that had very visible effects on the patient (Table 12, above). Two other activities-facility-based consultations and outreach-demonstrated moderate observable effects on patients, and school health activities did not have direct visible effect on school children.

### Summary of the results

The level of each of the five attributes of innovation was reported for each activity, and these results are summarised here. Each activity that scored "very high" for the activity scored a 5, and each activity that scored "high," "medium," "low", and "very low" were scored, respectively, as 4, 3, 2 and 1, based on the assumptions that the ratio between each category is the same and that each attribute has the same importance to describe an innovation. The sum of all the scores gave a total number of points for every activity. Table 13 summarises the measures of attributes for each activity by scoring every attribute from 5 to 1.

**Table 13:** Summary table of the attributes of every district activity

	**Relative advantage (5 = very high relative advantage)**	**Compatibility (5 = very highly compatible- 1 = very incompatible)**	**Complexity (5 = not complex – 1 = very highly complex)**	**Triability (5 = very highly triable – 1 = very highly untriable)**	**Observability (5 = very highly observable – 1 = very highly unobservable)**	**Total of points (max = 25)**
Facility-based Consultations	5	5	5	1	3	19
Cataract surgery	5	5	2	1	5	18
Outreach activities	5	2	2	1	3	13
School health	5	2	1	1	1	10

The activities that were the least sustained by increasing order were: school health (the least sustained), outreach activities, cataract surgeries, and facility-based consultations.

The results showed a relationship between the level of sustainability and the attributes of every activity. The activities with the lowest score for the attributes were less sustained. School health screening was the least sustained activity after the end of international funding. This activity also held the smallest score in terms of attributes: they were the most incompatible and most complex activities, as well as the least triable and observable activities, amongst the four district activities. In contrast, compared to the three other district activities, facility-based consultations were more likely to be routinised because they were perceived by the hospital managers as very compatible, and not complex.

## Discussion

The results indicated that differences in terms of sustainability varied in function of the type of activities and the attributes of these activities (i.e. observability, complexity, triability, and compatibility). Amongst the four district activities, facility-based activities (i.e. consultations and cataract surgeries) were maintained over time in a higher number of hospitals than cataract surgeries and outreach activities. School health screening was found to be the least sustained activity amongst the four district activities. School health activities were also scored as the most incompatible and most complex activity, and the least triable and observable activity amongst the four district activities. On the other hand, the facility-based consultations were more likely to be sustained because they were perceived by the hospital managers to be very compatible, and not complex, compared to the three other district activities. In other words, the four attributes of each eye care activity (i.e. incompatibility, complexity, triability, and observability) had an impact on their maintenance by hospital managers.

The relationships between attributes of activities and the continuation of these activities were explored in other studies. In terms of observability, Djellal and Gallouj [49] showed that the observable innovations such as new technology or new management systems determined the likelihood of their adoption by hospital staff. Innovation that showed visible benefits to its users were more likely to be adopted and maintained [30,31,50]. Singhal and Rogers [51] also showed that preventative health measures were less likely to be adopted and maintained than curative measures because they had less visible and immediate effects on an individual’s health than curative measures. Although all the studies mentioned above [30,49-51] were conducted in developed countries, their results were consistent with the results of the research presented here from a low income country, Ghana. Both school health and outreach activities were preventative. They had no direct impact on the health of the patient compared to consultations and cataract surgeries. From the patient and eye care staff perspectives, the observable effects of these two preventable activities were very limited. From the hospital managers’ perspective, school health and outreach activities had no financial observable added value. Both activities were delivered for free to the beneficiaries. Hospital managers were more likely to support facility-based consultations and cataract surgeries that had a positive observable impact on the population and the financial situation of the hospital.

In terms of complexity, past studies have shown that innovations were rejected by their health professionals if they were perceived by them, as users, as being too complicated to understand, use, or implement [28,30,52]. For example, the failure of the introduction of a shared electronic patient record system in the United Kingdom was explained by the high complexity of its utilisation [53]. Similar results were found in this study. In Ghana, the activities that were more likely to be stopped by hospital managers were those with the highest level of complexity: school health and outreach activities. These two activities involved a high number of actors and required efforts from the district hospital to inform and contact a wide range of actors who were for most of them from outside the health sphere (e.g. education authorities, school teachers, village leaders, sub-district nurses).

Regarding triability, several authors [31-33] found that innovations were adopted and maintained more easily if users could test them first before adopting them. Similar results were found in this study: activities that were the less triable (i.e., outreach activities and cataract surgeries) were more likely to be stopped as soon as the cessation of international funding.

In terms of compatibility, outreach activities raised numerous questions amongst hospital managers as to whether they should be run by hospitals or district authorities. The lack of clarification on this matter pushed most hospital managers to cease these activities as soon as funding stopped and check with the district authorities about who should run and fund these two activities. The mandate of district hospitals was to deliver facility-based services and serve the population living in their catchment area (i.e. the district). Every facility-based activity, such as consultations and cataract surgeries, was considered by hospital managers as fully compatible with the mandate of a Ghanaian district hospital. These two activities were fully in compliance with the mandate of district hospitals and no modification was required from the district hospitals to adjust their structure to deliver these activities. As such, facility-based consultations and cataract surgeries were perceived by hospital managers to be highly compatible innovations. The costs of these activities were included in the budget of the district hospital and were even refunded by National Health Insurance Scheme put in place by the Ministry of Health.

Hospital managers acknowledged that confusion existed over who should be responsible for the management and financing of outreach and school health activities. One hospital manager explained "Outreach activities should have been carried out the district authorities not by the hospitals. They are the ones in charge of all the health interventions that take place outside the walls of this hospital. However, they never took the initiative and our staff had to conduct the outreach consultations". As another hospital manager described it, "outreach activities and school health activities had never been seen as a problem as long as the costs of these activities were fully covered by the international organisation. As soon as the international organisation ceased its financial support, it became unclear for the health actors who should continue these activities, the hospital or the district authorities". The lack of clarity regarding the outreach activities and school health activities created conflicts between two distinct entities: the district health management teams (DHMTs) and the district hospital. In the Ghanaian health system, district hospitals are not accountable to the DHMTs. DHMTs are in charge of all the activities conducted at the primary health care level of the health system. Outreach consultations and school health activities were perceived as inconsistent with the mandate of the district hospitals. They also required slight adaptations from district hospital teams as described by one ophthalmic nurse: "outreach activities and school screening introduced new ways of working at the hospital. We had to contact headmasters or local authorities to offer our services. We then organised trips in collaboration with these people. This was very unusual for us. We were used to wait for the patient to come to the hospital. Now we had to do the inverse". Outreach and school activities were described as almost inconsistent with the mandate of hospitals and required adjustments from hospitals.

Several studies also showed that innovations that were compatible with individuals’ values [28-30], and organisations’ mandates were more likely to be maintained, as corroborated in the work presented in this paper.

As shown earlier, the results of this study were consistent with the results found in past studies [28-30,49,53-59]). The study in Ghana, however, presented two novel features:

i) First, the relationship between attributes and continuity of activities was established for the first time in a low-resource context; and

ii) Second, it is equally important to consider the individual parts (e.g., activities) of a health programme as it is to consider the programme as a whole. A health programme can be described as a set of activities. Disentangling projects into distinct activities allows sustainability to be assessed at a finer level, and helps identify which activities are likely to be maintained and which activities are more likely to stop.

This brings a new approach to evaluating sustainability, as well as providing new perspectives for implementing and funding agencies in their approaches to project implementation and sustainability. The implication is that the maintenance of health programmes can only be achieved through the introduction of customized strategies for each activity.

One limitation of the approach is that it does not capture the dynamics of health systems. The assessment at one point in time should be repeated over time to measure how the continuation of activities evolve and how the perception by health professionals in relation to attributes of innovations changes with the changing policy and finance environment. However, the utilisation of indicators validated by key informants was considered by the authors as appropriate way of describing the characteristics of innovations. Applications could be numerous in the field of international health to manage and predict implementation and scaling up of innovations (e.g. m-health, new drugs).

## Conclusions

Eye care activities that were more likely to be dropped were the ones that had the least observable effects, were the most complex and the least compatible with the mandate and financing system of the hospital. In the case of the eye care programme in Ghana, the first activities to be stopped were school screening and outreach consultations. Using diffusion of innovations theories can help predict the sustainability of specific activities within a health programme. The study also highlighted the need for disentangling the various components of a health programme in order to identify which activities are more likely to be continued within a health system. The same methodology could be used in a different setting and could help predict which innovations are more likely to be adopted and maintained over time in a hospital setting in a Western country, for example.

## Competing interests

The authors declare that they have no competing interests.

## Authors’ contribution

KB and PJ designed the methodology and the tools. KB collected data. KB and PJ analysed data. KB wrote the manuscript. PJ edited the manuscript. Both authors read and approved the final manuscript.

## Pre-publication history

The pre-publication history for this paper can be accessed here:

http://www.biomedcentral.com/1472-6963/14/77/prepub
